# Imaging Diagnosis of Hepatocellular Carcinoma: A State-of-the-Art Review

**DOI:** 10.3390/diagnostics13040625

**Published:** 2023-02-08

**Authors:** Gianvito Candita, Sara Rossi, Karolina Cwiklinska, Salvatore Claudio Fanni, Dania Cioni, Riccardo Lencioni, Emanuele Neri

**Affiliations:** Department of Translational Research, Academic Radiology, University of Pisa, 56124 Pisa, Italy

**Keywords:** hepatocellular carcinoma, computed tomography, ultrasound, magnetic resonance imaging, artificial intelligence

## Abstract

Hepatocellular carcinoma (HCC) remains not only a cause of a considerable part of oncologic mortality, but also a diagnostic and therapeutic challenge for healthcare systems worldwide. Early detection of the disease and consequential adequate therapy are imperative to increase patients’ quality of life and survival. Imaging plays, therefore, a crucial role in the surveillance of patients at risk, the detection and diagnosis of HCC nodules, as well as in the follow-up post-treatment. The unique imaging characteristics of HCC lesions, deriving mainly from the assessment of their vascularity on contrast-enhanced computed tomography (CT), magnetic resonance (MR) or contrast-enhanced ultrasound (CEUS), allow for a more accurate, noninvasive diagnosis and staging. The role of imaging in the management of HCC has further expanded beyond the plain confirmation of a suspected diagnosis due to the introduction of ultrasound and hepatobiliary MRI contrast agents, which allow for the detection of hepatocarcinogenesis even at an early stage. Moreover, the recent technological advancements in artificial intelligence (AI) in radiology contribute an important tool for the diagnostic prediction, prognosis and evaluation of treatment response in the clinical course of the disease. This review presents current imaging modalities and their central role in the management of patients at risk and with HCC.

## 1. Introduction

Liver malignancies undoubtedly represent a global health challenge, with an estimated annual incidence of more than one million cases in 2025 [1]. Primary liver cancer is the sixth most commonly occurring cancer in the world and the third largest contributor to oncologic mortality [1].

Hepatocellular carcinoma (HCC) accounts for a great majority of liver cancer diagnoses and deaths [2].

Although hepatitis B virus (HBV) and hepatitis C virus (HCV) remain the most important global risk factors worldwide, their impact on the rise of HCC will decline in Western countries due to the availability of increasingly efficient antiviral therapies and preventive policies [3]. As overweight will become endemic worldwide, non-alcoholic fatty liver disease (NAFLD) is likely to become the major contributor to the epidemiology of HCC in the coming years, with a higher risk of incidentally detecting large liver nodules also in younger asymptomatic patients [4]. Other established risk factors of HCC are alcohol consumption [5] and idiopathic liver diseases (e.g., hemochromatosis or primary sclerosing cholangitis) [6].

As a result of several studies on HCC pathology published in the past years, hepatocarcinogenesis is well established nowadays. In cirrhotic livers, metabolic and oxidative insults cause an increased turnover of hepatocytes with a progressive accumulation of genetic mutations [7]. Notably, during the progression from cirrhotic nodules through dysplastic nodules and early HCC to advanced HCC, portal tracts progressively diminish, whereas newly formed unpaired arteries develop due to the tumoral release of vascular endothelial growth factor (VEGF) [7]. Therefore, HCC nodules present a more notable arterial supply as compared to the healthy surrounding parenchyma with the typical greater supply from the portal vein.

Among all the tested serum biomarkers, alpha-fetoprotein (AFP) has proven to improve diagnostic efficiency and to be useful in the evaluation of treatment response in patients with HCC [8].

Unfortunately, the prognosis of patients with HCC remains poor thus far, with an overall ratio of mortality to incidence of 0.91 [9]. However, the accelerated introduction of novel therapeutic modalities is expected to lead to a more favorable scenario. Indeed, due to the recent advances in the oncologic armamentarium, the Barcelona Clinic Liver Cancer (BCLC) treatment strategy was updated in 2022, including the latest evidence of promising medical and interventional therapies [10].

As a matter of fact, in patients at risk, surveillance plays a pivotal role in the detection of small HCC nodules, whose treatment may consist of less invasive and more effective therapies (e.g., percutaneous thermal ablation, surgical excision) [11].

As stated by the latest clinical practice guidelines, published by the European Association for the Study of the Liver (EASL) [12] in 2018, HCC is unique among other cancers in showing typical characteristics on contrast-enhanced computed tomography (CT), magnetic resonance imaging (MRI) or contrast-enhanced ultrasound (CEUS), thus allowing for a highly accurate diagnosis of HCC in patients with cirrhosis. As a result, mini-invasive percutaneous imaging-guided biopsy is strongly recommended for liver nodules with an atypical contrast enhancement [13] or in non-cirrhotic patients [14].

The ability of cross-sectional imaging studies to reliably detect and diagnose HCC in the cirrhotic liver relies primarily on characterizing the enhancement of a suspected lesion as compared to the background liver parenchyma in the hepatic arterial, portal-venous and subsequent phases. The abovementioned differences in the blood flow and extracellular volume between HCC tissue and non-neoplastic cirrhotic liver tissue result in the hallmark imaging characteristics of HCC during the multiphasic flow of contrast, including arterial phase hyperenhancement, subsequent wash-out appearance and capsule appearance [15].

CEUS is a dynamic imaging technique, able to assess the contrast-enhancement pattern of liver nodules in real time, with a considerably higher temporal resolution than that possible to obtain with CT and MRI [16]. CEUS, however, presents some important drawbacks. First of all, CEUS is not a cross-sectional imaging modality, thus not allowing for the detection of distant nodules not seen or included by the operator in the scan after contrast injection. Moreover, ultrasound (US) examination is an operator-dependent modality and may be limited in the detection of nodules in overweight patients or nodules with a difficult location [17].

MRI offers a number of detailed imaging sequences, including T2-weighted and diffusion-weighted images, which may help in the detection of suspicious nodules, although baseline images rarely provide sufficient specificity to enable noninvasive diagnosis. Furthermore, in recent years, two liver-specific contrast agents (gadobenate dimeglumine and gadoxetic acid) have shown to improve the detection of even relatively small and subtle lesions with a hypointense appearance in the hepatobiliary phase [18].

Nevertheless, MRI has some important diagnostic disadvantages, including less availability, greater technical complexity, higher susceptibility to artifacts, higher costs and less consistent image quality. In particular, MRI quality may be compromised in patients with difficulty in breath-holding, trouble keeping still, or large-volume ascites. MRI permits a locoregional evaluation of parenchyma and nodes in the upper abdomen without any information on possible distant metastases. For these reasons, the comparative diagnostic performance of a multiphasic CT and an MRI in real-life practice remains uncertain [19].

In the recent years a rising interest in artificial intelligence (AI) has been observed, and, undeniably, oncologic imaging is one of the most empowered application fields [20,21,22]. Machine learning (ML) is a branch of AI that focuses on the development of computer algorithms able to learn from structured data to make predictions on decisions without being explicitly programmed to do so. In the oncologic imaging setting, ML is usually combined with radiomics, defined as the process of extracting high-dimensional quantitative features from medical images [23,24,25]. However, radiomic pipeline consists of numerous steps characterized by several factors, leading to a significant variability between studies affecting their repeatability [26,27].

To overcome the need of prior feature extraction, deep learning (DL) algorithms were developed. DL is a subfield of ML using an artificial neural network (ANN) and has achieved very optimistic performance in image analyses.

Radiomics-based ML and DL have already demonstrated great potential in the diagnosis, staging, survival prediction and tumor response control of HCC [28].

## 2. Ultrasound

Liver cirrhosis is, thus far, the primary risk factor for HCC, with affected patients requiring periodical imaging surveillance. US is a perfect choice for this purpose due to its safety, wide availability, cost-effectiveness and accuracy in detecting focal liver lesions (FLLs). Once a FLL is detected, US can assist in its characterization using different ultrasonographic techniques, including B-mode, color- and power-Doppler techniques and CEUS [29].

The appearances of HCC nodules on US vary depending on the size and degree of differentiation. The lesion margins are usually relatively well circumscribed in the nodular type but poorly defined in the massive type [30]. HCC nodules smaller than 10 mm are almost hypoechoic or isoechoic, with low-level internal echoes that increase with tissue cellularity. When tumor growth occurs, fatty change is most frequently observed at a tumor diameter of 10–15 mm, and the internal echoes of such nodules are hyperechoic [31]. In HCC nodules greater than 20 mm, typical US patterns such as the “mosaic pattern”, “nodule-in-nodule appearance”, “peripheral sonolucency” (halo sign) and “lateral shadow” can be more commonly recognized [32].

The evaluation of intranodular vascularity may play a key role in the characterization of FLLs. For this purpose, color Doppler is typically the first-line modality of assessment, even though it encounters different technical limitations such as Doppler angle dependence, operator dependence, low sensitivity to slow flow and overwriting artifacts [30]. Usually, once the tumor increases in size, the “basket” pattern, referring to the presence of a fine network of arterial branches surrounding the lesion, can be appreciated [33]. Using spectral analysis, both pulsatile and continuous waveforms can be recorded, which correspond to the arterial and venous origin of blood supply, respectively. In massive-type HCC, an overall irregular pattern of vascularity, can be appreciated. As a general rule, a continuous portal-like waveform indicates a dysplastic nodule or a well-differentiated HCC; contrarily, a pulsatile arterial waveform is suggestive of advanced HCC [30].

Due to the fact that worldwide ultrasound represents the imaging modality of choice in surveilling patients at risk, the introduction of the US LI-RADS^®^ (Liver Imaging Reporting and Data System), a US-based classification system, was issued by the American College of Radiology in 2017 [34]. Evaluating the size and echogenicity, this system assesses the quality of examination and the potential of a FLL to represent HCC and suggests further management [35].

US, in general, has a reported sensitivity of 98% and specificity of 85% for overall HCC detection. Tumor size is nonetheless a significant factor as the technique’s sensitivity reaches approximately 65% for lesions <2 cm [36].

The introduction of CEUS in the evaluation of FLLs certainly represented a turning point in the ultrasonographic diagnosis of HCC. US contrast agents (USCAs) consist of different generations of intravascular gas microbubbles with specific nonlinear acoustic properties [37]. After bolus intravenous injection, USCA allows capillary blood flow to be imaged and contrast enhancement to be assessed, with a much higher temporal resolution compared to CT and MRI [16]. CEUS has proven to be a safe procedure, with low clinical reactions to USCAs reported in the literature and few absolute contraindications (e.g., severe coronary artery disease, pulmonary hypertension). Several studies have stated that CEUS has a significant role as a problem-solving imaging technique for detection of perfusion abnormalities in patients with renal failure and/or at high risk of adverse reaction to CT or MRI contrast agents [17].

**Figure 1:**
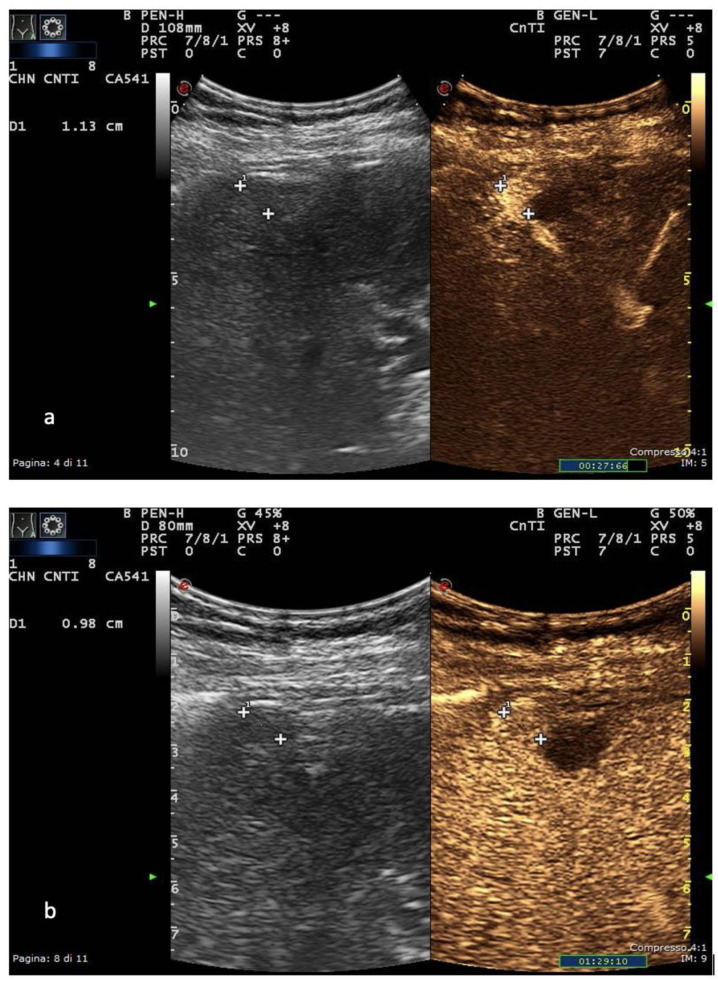
US and CEUS surveillance examination in a patient with HBV-related cirrhosis. Baseline images detect the presence of a centimetric subcapsular hypoechoic nodule. After administration of USCA, the lesion shows arterial hyperenhancement (**a**) with a mild portal-venous wash-out (**b**).

In Europe, CEUS is usually performed with SonoVue^®^ (Bracco, Milan, Italy), which is not uptaken by Kupffer cells and hence produces an arterial, portal-venous and late phase [38]. The hallmark of HCC on CEUS using SonoVue^®^ is a homogeneous and intense arterial phase hyper-enhancement (APHE) with mild wash-out starting >60 s after injection [39] (Figure 1).

The timing and degree of wash-out are important for the characterization of HCC, which typically shows milder hypo-enhancement compared to metastasis and cholangiocarcinoma. Nodules measuring >5 cm may show heterogeneous enhancement due to necrosis. Both the size and the degree of differentiation affect the enhancement pattern of HCC [40]. Wash-out is less often seen in HCC nodules <2 cm but is more frequent in HCC with poorer grades of differentiation [41].

On the other hand, Sonazoid^®^ (GE Healthcare, Amersham, UK) is a second-generation USCA whose clinical usage was approved in Japan, South Korea and China. As opposed to Sonovue^®^, Sonazoid^®^ is uptaken by Kupffer cells and produces a late homonym phase in which HCC nodules appear as hypoechoic lesions as compared to the surrounding parenchyma [42].

Moreover, a CEUS LI-RADS^®^ [43] algorithm has been introduced by the American College of Radiology to aid in the accurate characterization of nodules in liver cirrhosis patients. The major criteria are APHE, nodule size and portal-late mild wash-out. A rim APHE and an early (<60 s) or marked wash-out represent LI-RADS M criteria (LR-M), favoring the diagnosis of a non-hepatocellular malignancy [43].

## 3. Computed Tomography

Nowadays, Multidetector Computed Tomography (MDCT) plays a key role in the diagnostic management of cirrhotic patients who are at an increased risk of developing HCC. According to the majority of guidelines, recognition of a nodule ≥10 mm by ultrasonography (US) during HCC surveillance should be followed by a contrast-enhanced CT or MRI examination [44].

MDCT is actually a widely available and rapid imaging modality. Most modern CT scanners have the capability to capture images with wide-detector arrays, typically more than eight-row detectors, allowing for high spatial resolution. Premium CT scanners offer even wider detector arrays with up to 320 detector rows that cover up to 16 cm in the *z*-axis and fast gantry rotation times down to 0.25 s [45].

As compared to MRI, MDCT is a faster and better-tolerated examination, less prone to motion artifacts, particularly useful in non-cooperative patients or in those who are unable to hold their breath. The main disadvantages of MDCT include radiation exposure and relatively low contrast resolution of tissue, even though iterative reconstruction models have further enabled radiation dose reduction by reducing CT image noise [30].

The baseline pre-contrast phase examination serves as a baseline for determining the extent of liver lesion and is useful to assess background liver disease such as steatosis or cirrhosis [46] For HCC evaluation, the non-contrast phase helps identify subtle areas of arterial phase hyperenhancement and is essential to distinguish hyperdense lipiodol staining and blood products in patients who previously underwent intra-arterial or percutaneous treatments [47].

However, multiphase contrast-enhanced CT and/or MRI examinations consisting of the late arterial, portal-venous and delayed phase are essential for a confident imaging diagnosis of HCC [48].

**Figure 2:**
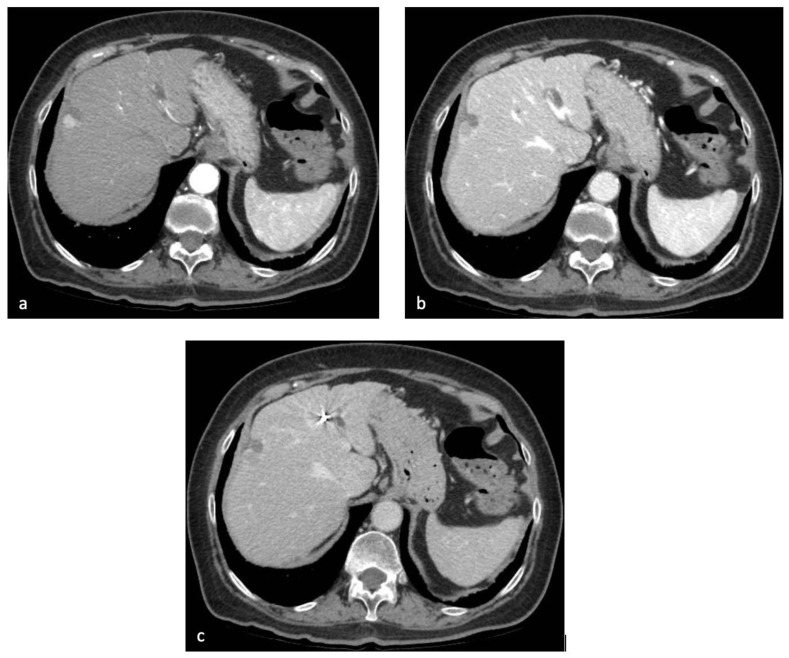
Contrast-enhanced CT of the upper abdomen in the patient discussed in Figure 1. After administration of iodinated contrast agent, the subcapsular lesion showed arterial hyperenhancement (**a**), with progressive wash-out in the portal-venous (**b**) and delayed (**c**) phase.

Whereas the portal-venous phase is sufficient for the detection of hypovascular liver metastases, the late arterial and delayed phases are most important for the evaluation of hypervascular tumors including HCC (Figure 2).

The typical hallmark diagnostic feature of HCC is the combination of non-rim APHE on the late arterial phase and non-peripheral wash-out appearance on the portal-venous and/or delayed phases, thereby reflecting the peculiar vascular derangements induced by hepatocarcinogenesis [49].

As stated by different current guidelines [12], the late hepatic arterial phase (35 s) is considered the most consistent vascular phase for the assessment of HCC, as APHE is an essential finding in making a definitive imaging diagnosis of HCC [50]. The late arterial phase should be characterized by full hepatic arterial enhancement with good portal vein enhancement, but no antegrade enhancement of the hepatic veins. As some HCCs are not conspicuous until the late hepatic arterial phase, earlier arterial phase imaging can result in reduced sensitivity [51]. Moreover, as a favorable late arterial phase occurs during a restricted time interval, individualized CT scan protocols (e.g., test-bolus, bolus-tracking) are recommended.

The portal-venous phase (70–80 s) occurs when enhancement of the portal and hepatic veins is higher and there is peak parenchymal enhancement of the liver. Portal-venous phase FLL imaging best demonstrates the “wash-out appearance” due to the peak enhancement of the surrounding liver [52]. The detection of peripheral washout on the portal phase is not specific for HCC nodules, as intrahepatic cholangiocarcinoma may also show this kind of appearance [53].

The delayed phase (3–5 min) is acquired when overall vessel brightness decreases as compared to the portal-venous phase. A combination of the portal-venous phase and delayed phase can more reliably demonstrate the “wash-out appearance” and “capsule appearance” of the HCC nodule [53]. Conversely, cholangiocarcinoma typically shows peripheral enhancement in the arterial phase, with centripetal progressive reinforcement in the delayed phase [54].

The detection of an “enhancing capsule” [55], with the appearance of a uniformly thick enhancement at the peripheral rim of the nodule on the portal and delayed phase, is another major criterion included in the LI-RADS. The tumor capsule is detected in about 70% of HCCs and is a pathologic feature of progressed disease [30].

Apart from the major imaging features, the LI-RADS CT/MRI contains many ancillary features, including nodule-in-nodule architecture, mosaic appearance and non-enhancing capsule, that may favor the diagnosis of HCC [34].

The nodule-in-nodule architecture consists in the detection of a progressed HCC within a dysplastic nodule or an early HCC. The inner nodule shows APHE, while the parent nodule appears hypo- or iso-attenuated. The nodule-in-nodule appearance presents a poor prognostic value, as the inner hyper-enhancing nodule has a short volume-doubling time [56].

Similarly, the mosaic appearance is the result of a presence of areas within larger nodules in various steps of dedifferentiation. On imaging, similar nodules are composed of compartments with variable enhancements, separated by irregular enhancing septa and necrotic areas [57]. The mosaic pattern is observed in 28–63% nodules of HCCs [30].

The non-enhancing capsule refers to a capsule appearance that is constantly hypodense on dynamic CT/MRI examinations [58].

In recent years, dual-energy CT (DECT) has become increasingly available. DECT can acquire two sets of images of the same tissue using different photon spectra (high and low kVp). By adjusting the photon spectrum, the optimal single energy with an optimized contrast-to-noise ratio (CNR) can be obtained, which, in turn, improves the detection rate of smaller tissue density differences as well as small lesions [59]. As compared to low kVp CT scans, at an equal radiation dose [60], DECT showed higher CNR of HCC and higher image quality, thus allowing the radiologist to evaluate small lesions that were not detectable on conventional CT scan [61].

Furthermore, recently, CT liver perfusion (CTLP) has emerged as a useful imaging modality for quantitative evaluation of tumor angiogenesis. CTLP is based on the analysis of a dataset that includes sequential CT images of the liver acquired over time following intravenous contrast injection, thus measuring the change of attenuation of regions of interest within the liver parenchyma [62]. Conventional CT might mischaracterize small HCC nodules without a clear APHE; CTLP can separate the hepatic arterial from the portal-venous component of blood flow in order to identify the nodules with a still incomplete neo-angiogenesis [63].

In the setting of HCC, CTLP demonstrated fair diagnostic accuracy in the first diagnosis [64] and in assessing treatment response through the evaluation in the arterial perfusion changes [65].

## 4. Magnetic Resonance Imaging

The introduction of MRI in clinical practice has radically changed the diagnostic algorithm of HCC, since it may achieve a higher contrast resolution and is able to characterize more tissue properties other than tissue density and vascularization [66]. According to recent meta-analyses, the pooled overall sensitivity and specificity of contrast-enhanced MRI are 70% and 94%, respectively, in the detection of HCC nodules [67]. Nevertheless, sensitivity is greater for lesions >2 cm (almost 100%) but drops to 60% for lesions smaller than 2 cm, and it is even lower for lesions smaller than 1 cm [68]. Therefore, MRI has proven to outperform CT for the diagnosis of HCCs smaller than 2 cm, with comparable accuracy for lesions ≥2 cm [30]. For this reason, MRI is also a useful imaging modality in the surveillance of cirrhotic patients at risk. Nowadays, a prompt diagnosis of small nodules is mandatory to assure a radical treatment, thus augmenting overall survival [69].

As stated before, large HCC nodules generally show the typical imaging hallmarks (“wash-in/wash-out” appearance) that enable a radiologist to make a definitive diagnosis also in gadolinium-enhanced MRI examinations. However, APHE may not be present in a large percentage of early and poorly differentiated HCCs, which should not be definitively assessed according to the current guidelines [15]. In such cases, MRI plays an indisputable role in finding out the presence of ancillary features in differently weighted images, keeping in mind that lesions <1 cm cannot be definitively characterized as HCC and follow-up is advised [30].

According to LI-RADS, the detection of a capsule is a major finding typically found in progressed HCCs. HCC capsules usually show low T1 and T2 intensity, with a mild enhancement in the portal-venous and delayed phases and are thicker than cirrhotic fibrotic septa [58]. The detection of a disrupted capsule is a negative prognostic factor, as a higher recurrence rate after surgical or interventional treatment is reported [55].

Most large HCCs show moderate hyperintensity on T2-weighted (T2-w) sequences, probably due to a higher cellularity, an increased arterial blood flow and a decreased portal vascularity [70].

Conversely, dysplastic nodules and early HCCs appear iso- or hypo-intense as compared to the background liver [71]. However, mildly increased T2 signal intensity is not a specific imaging feature as it is also imaged in other malignant lesions of the liver [72].

On the other hand, hyperintensity on T1-weighted (T1-w) sequences may be detected if a high amount of fat or glycogen is present within the HCC nodule.

Almost 40% of early HCCs present with intranodular fatty changes, which tend to regress during the tumoral progression to higher histological grades [73]. On chemical shift sequences, fatty areas within the nodule show the characteristic signal drop on the opposed-phase compared to in-phase [74]. Glycogen may be present as a result of the hypercellularity within the nodule [75] and does not show signal drop on chemical shift sequences.

Furthermore, MRI is the preferred imaging modality in surveilling patients with hemochromatosis (liver iron overload), which is itself a risk factor for HCC development [76]. Iron-rich nodules usually appear hypointense on T1-w images and moderately to markedly hypointense on T2-w and T2*-w images [75]. In such parenchymal background, iron-free nodules appear as hyperintense on T1-weighted images and are highly suspicious for a dysplastic or HCC lesion [77].

Since hyperintensity on T1-weighted baseline sequences may produce misinterpretation, subtraction techniques are always recommended in order to correctly detect APHE [78].

In recent years, diffusion-weighted imaging (DWI) has emerged as a baseline MRI sequence that evaluates the reduced diffusivity of water molecules among the closely packed cells within HCC nodules [79]. In general, higher histological grades are associated with higher DWI signal and corresponding lower apparent diffusion coefficient (ADC) values. Early HCCs may be misdiagnosed on DWI due to their relatively low cellular density [80]. However, a restricted diffusion is an ancillary feature that favors the diagnosis of liver malignancy, but it is not specific for HCC [81]; evaluating the appearance on different MRI sequences, including contrast-enhanced images, may support the diagnosis of HCC. DWI is useful in corroborating the suspicion in typical and atypical HCC nodules or in patients that cannot undergo intravenous contrast injection (e.g., for a previous allergic reaction), thus increasing the overall sensitivity of HCC detection [82].

In the last decade, several meta-analyses have established that MRI paired with gadoxetic acid-based hepatobiliary contrast agents presents a higher sensitivity than MRI paired with extracellular agents, in particular in the setting of small HCCs that may not show the typical APHE [83]. Hepatobiliary contrast agents (gadobenate dimeglumine, gadoxetate disodium) are selectively taken up by normal hepatocytes through specific organic anion transporting polypeptide (OATP) transporters, allowing the acquisition of hepatobiliary phase (HBP) images at 20–40 min [84]. Nodules with a lack of hepatocytes (angiomas) or degenerated hepatocytes lacking OATP (malignancies) are hypointense on HBP [85], while lesions with a higher number of functioning hepatocytes (focal nodular hyperplasia, low-grade dysplastic nodules) may appear hyperintense on HBP [86].

**Figure 3:**
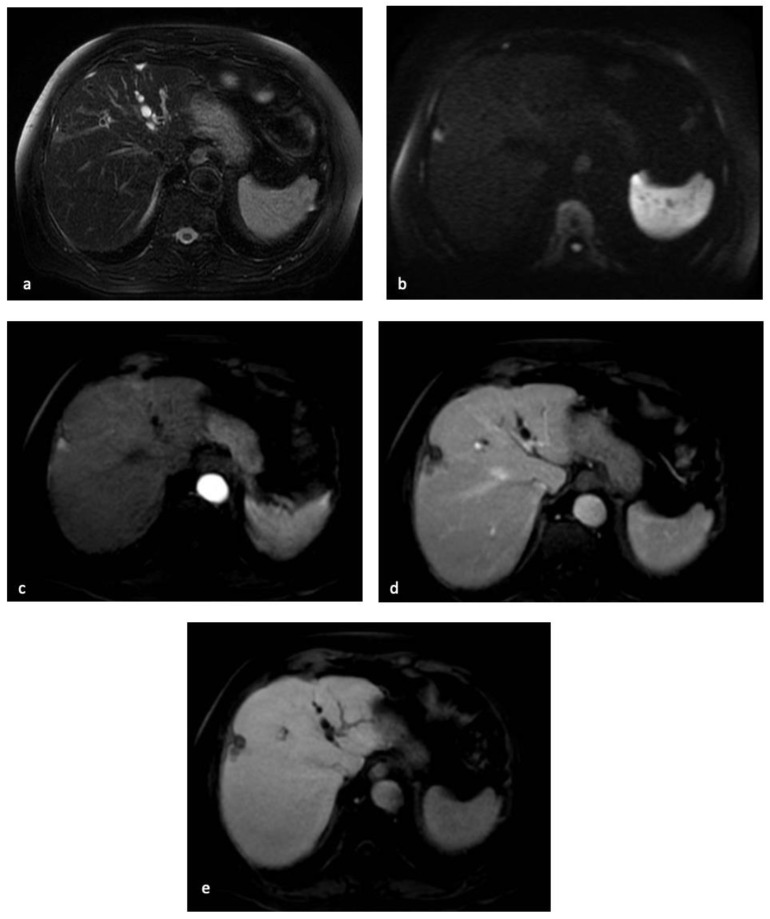
MR examination of the patient discussed in Figure 1 and Figure 2. On T2-weighted images, the centimetric subcapsular appeared as hyperintense (**a**). On DWI with a b-value of 1000, the lesion showed signal restriction (**b**). After administration of a hepatobiliary contrast agent, the lesion showed arterial hyperenhancement (**c**) with hypointensity in the portal-venous phase (**d**) and in the hepatobiliary phase. (**e**) The lesion appeared hypointense.

Since up to 90% of HCCs demonstrate hypointensity in the HBP, this ancillary feature may contribute to the differentiation of HCC from benign nodules developed in chronic liver diseases (Figure 3) [87].

However, until now, there has been no established consensus regarding the value of HBP hypointensity during liver MRI. In East Asia, some guidelines attribute importance to the use of HBP hypointense appearance, thus permitting the diagnosis of smaller HCCs [88]. Meanwhile, in the Western countries, where liver transplantation is one of the major treatment options [89], the practice guidelines suggest that wash-out should be determined in the portal phase, thus obtaining the highest specificity [12]. In fact, recent studies have suggested that HBP hypointense appearance is highly sensitive and specific for HCC when combining with non-rim APHE [84].

In addition, in MRI, perfusion imaging is a quantitative technique that provides information about tissue microcirculation. In the liver, the most used approach is dynamic contrast-enhanced (DCE) MRI, which requires gadolinium contrast administration as a tracer, followed by consequential acquisition of signal-time curves that quantify changes in contrast concentrations over time [90]. DCE consists of free-breathing 3D perfusion sequences covering the entire liver with a short acquisition time (1–2 s) repeated for up to 5 min after contrast administration [91].

DCE-MRI provides information based on the intralesional temporal distribution of contrast agents in lesions that often present with a heterogeneous vascular network. Time-to-peak enhancement (time between arrival of the tracer and maximum enhancement), area under the curve (amount of enhancement during a specific time interval), maximum enhancement (peak height) and maximum slope are semi-quantitative analyses affected by acquisition parameters, injection protocols and the patient’s physical conditions [92]. On the other hand, true quantitative models evaluate the change in concentrations of the contrast agent using pharmacokinetic modeling techniques [93].

## 5. PET/CT

Though HCC diagnosis is primarily based on the typical characteristics of contrast hyperenhancement and wash-out on CT and MRI, some of the biologic features of HCC can be appreciated fully only with the 18F-fluorodeoxyglucose positron-emission tomography (FDG-PET)/CT. This imaging modality provides some additional information on primary HCC lesions and extrahepatic metastases which aids clinicians with treatment selection [94]. FDG-PET/CT is an extremely useful tool in the evaluation of many oncologic patients, yet it is not routinely used for HCC as it is limited by low sensitivity due to the high physiologic uptake of liver tissue and the variable expression of glucose transporters and glycolytic activity in HCC nodules [95]. In fact, FDG usually accumulates in poorly differentiated HCCs but not in well-differentiated ones. Furthermore, since a poorly differentiated HCC is more likely to metastasize, FDG-PET/CT may be useful to detect distant metastasis and complete the staging in uncertain cases [96].

However, tracers based on choline recently showed improved detection rates of well-differentiated HCCs [97]. Dual-tracer PET/CT combining choline and FDG as tracers has shown high overlap between well- and less-differentiated HCCs, thus making it possible to classify lesions in proliferative (poorly differentiated nodules) and non-proliferative (well-differentiated nodules) [98].

FDG-PET/CT can be used to monitor treatment response and provide prognostic information on the risk of HCC recurrence after surgery or interventional treatment, as the scans reflect high tissue metabolism that may be indicative of recurrent disease even in areas of increased tissue rearrangement due to the treatment [94].

## 6. Artificial Intelligence

Artificial intelligence (AI) represents the ability of machines to emulate the intelligence of human beings [99]. Radiomics-based ML and DL could potentially assist radiologists in HCC imaging by overcoming some of the main limitations presented by imaging modalities that were described above. Indeed, the human eye, especially with low expertise, could lead to wrong or indefinite diagnoses, leading to several other investigations with various modalities. This is particularly true in US imaging, which hugely relies on the radiologist’s expertise and which represents the primary technique used to follow-up patients suffering from liver cirrhosis—remaining one of the principal risk factors for HCC development. Indeed, AI could empower the role of US imaging, being a safe, non-invasive and rapid modality; decreasing the use of second-level imaging techniques generally based on contrast media; and attenuating the limitations of US. The advantages of AI use for patients with HCC could be represented by the time reduction needed to identify the malignant lesion and, thus, faster treatment; its differential diagnosis between benign and malignant conditions to avoid unnecessary CT/MRI studies; and, finally, the ability of AI to differentiate HCC from other primary or secondary malignancies [100].

AI has already been demonstrated to reduce the time-to-diagnosis of HCC by US using ML and DL algorithms, with the latter characterized by superior accuracy, sensitivity and area under the curve (AUC) [101].

Regarding the differential diagnosis between benign entities (cysts or hemangiomas) and malignancies, Schmauch developed an artificial neural network (ANN) that achieved an AUC of 0.924 [102]. Accordingly, Guo et al. implemented a computer aided diagnosis (CAD) system for three-phase CEUS to differentiate between benign and malignant liver lesions and found an overall accuracy of 93.56 ± 5.90% [103].

HCC, as previously mentioned, can sometimes have characteristics of other malignant lesions, and differentiating between HCC and other primitive lesions or secondary ones may become challenging. AI may help in this setting, as demonstrated by Mao et al., who reported an accuracy of 0.843 ± 0.078 in differentiating between primary and metastatic liver cancer (AUC, 0.816 ± 0.088; sensitivity, 0.768 ± 0.232; specificity, 0.880 ± 0.117) [104].

Another challenge for radiologists is to differentiate HCC from cholangiocarcinoma, or a combination of the two (hepato-cholangiocarcinoma), as the two pathologies have the same risk factors and, especially in US imaging, lack particular characteristics to distinguish between them. Currently, AFP and carbohydrate antigen 19-9 are considered the ideal serum tumor markers for HCC and intrahepatic cholangiocarcinoma, yet they are generally deemed unsatisfactory in diagnostic sensitivity or specificity [104]. The two tumor markers are especially unreliable if the diagnosis is made based on them alone [1]. Ichikawa et al. determined the imaging hallmarks for distinguishing intrahepatic mass-forming biliary carcinomas from HCC, and the diagnostic value was further verified by Bayesian statistics (AUC = 0.960) [105].

US imaging is also suitable for radiomics-based approaches, and its utility has already been proven in distinguishing between low- and high-grade HCC. This differentiation is important to establish patients’ prognosis and to estimate the probability of recurrence or metastasis after treatment [106], especially because patients with high-grade HCC have poor prognosis. According to Ren et al., grayscale ultrasomics features can be used to distinguish high- and low-grade HCC with a *p* value of <0.05, providing information on tumor heterogeneity which cannot be identified by human eye in normal US images [107]. Radiomics-based model benefit from the combination with clinical data, as demonstrated by Wang et al., who combined radiomics features extracted from CEUS with clinical variables to improve the tumor grading performance.

The use of AI on CT images could enhance its diagnostic potential for HCC and aid differentiating its different aspects (i.e., nodular, diffuse or massive), as well as distinguish HCC from other benign and malignant liver lesions and estimate a grading scale.

Convolutional neural networks (CNN) are able to automatically perform liver and tumor segmentation and classify lesions as nodular, diffuse or massive type. Studies have demonstrated the superiority of this fully automated method over the semi-automated one [108].

As mentioned before, to distinguish HCC from other liver lesions, the use of contrast media aids the study of vascular patterns of different kinds of lesions. CNN is a potential method to diagnose and differentiate HCC using the Liver Imaging-Reporting and Data System (LI-RADS). The use of CNN can reduce radiation dose to patients because it is able to diagnose HCC based on a three-phase CT without the pre-contrast phase. In fact, this protocol shows similar diagnostic accuracy compared to the four-phase protocol, limiting the radiation dose to patients, especially as these patients need multiple CTs in the course of follow-up [109]. Radiomic-based ML could also assist radiologists in diagnosing HCC when it shows indeterminate or doubtful aspects without the specific wash-in and wash-out imaging features [110]. It is based on different tumor aspect during arterial and portal phase, such as, for example, wash-out without a clear wash-in. This technique is used for images taken with different protocols, so it can be used for images taken at different institutes. Nevertheless, the features extracted often overlap between HCC and other malignant lesions. This is a limit of radiomics that is able to well differentiate benignity from malignity, yet may not always identify the malignant lesion as HCC.

AI could also help to estimate patients’ prognosis, evaluating, for example, the recurrence risk or microvascular invasion (MVI) tumor pattern. Studies have shown that MVI is an independent histopathological prognostic factor associated with survival in all-stage HCC patients [111]. MVI has been reported to be a better predictor of tumor recurrence and overall survival than the Milan criteria [112]. Patients with a poor prognosis need a more aggressive treatment approach. Different features are evaluated to distinguish MVI, such as the smooth and irregular margin of lesions, presence of internal tumor arteries, hypodense halo, peritumoral enhancement and lobes involved. In the study published by Jiang et al., the median recurrence-free survival (RFS) of the entire cohort was 22 months while the RFS of patients with MVI was 6 months, and a CNN was able to accurately differentiate MVI pre-operatively [113].

MVI invasion is also important to evaluate recurrence risk after trans-arterial chemoembolization [114].

Studies on AI and MRI are still limited compared to US and CT. AI in MRI can differentiate LI-RADS 3 grade from LI-RADS 4-5, which is extremely important for clinical decision and patient management. In fact, LI-RADS 3 needs no or less invasive management. Many LR-3 lesions are benign hyper-enhancing pseudolesions which can be followed for stability with imaging, whereas 80% of biopsied LR-4 lesions are HCC, and 68% of untreated LR-4 lesions become LR-5 lesions within two years [50]. LR-4 lesions may be biopsied, while an LR-5 score indicates HCC diagnostic certainty and biopsy is usually not needed before treatment [115].

The results demonstrated that tumor size and shape, associated with its contrast aspect, are important factors for HCC diagnosis. In addition, it is demonstrated that the late contrast phase does not contribute to the LI-RADS classification performance of CNN model and it can be avoided [116]. This condition makes it possible to reduce time for MRI imaging, limiting patients’ artifacts. CNN improves the recognition of this classification and reduces misdiagnosis by radiologists.

## 7. Conclusions

Imaging plays a pivotal role in the multidisciplinary management of patients at risk or suffering from HCC and in the radiological evaluation of response to treatment.

US is the most recognized imaging modality for HCC surveillance, even though MRI has been recently proved to be a useful tool in surveilling cirrhotic patients.

However, non-invasive diagnosis of HCC mainly relies on CT and MR examination. Different radiological hallmarks have been described, with APHE being an essential finding in making a definitive diagnosis of HCC. The recent introduction of hepatobiliary contrast agent in liver MRI has shown to increase sensitivity and specificity in assessing HCC nodules, as well as in the absence of typical APHE, and may change the diagnostic imaging algorithm in the coming years.

Furthermore, recent applications of AI, including radiomics and machine learning, have shown interesting results in the setting of liver imaging in patients with HCC. AI has proven to empower the role of imaging diagnosis, helping the radiologist to distinguish HCC from other liver malignancies in atypical or doubtful cases or to evaluate microvascular invasion that heavily modify patients’ prognosis. Through AI applications, it will be reasonably possible in the upcoming years to reduce the time and number of examinations needed to characterize malignant lesions, thus allowing for faster diagnosis, better prognosis and reduced medical costs.

## Data Availability

Not applicable.
